# Comparison of Intramedullary Nails with Cephalic Screws and Dynamic Hip Screw in the Treatment of Unstable Intertrochanteric Fractures in Adults – A Systematic Review and Meta-Analysis

**DOI:** 10.1055/s-0045-1812023

**Published:** 2025-11-18

**Authors:** João Protásio, Paulo Victor Dias Almeida, Mariana Garcia Martins Castro

**Affiliations:** 1 Hospital Geral de Palmas, Universidade Federal do Tocantins, Palmas, TO, Brazil; 2 Palmas Municipal Heath Department, TO, Brazil

**Keywords:** bone nails, fractures, bone, hip fractures, intramedullary nailing, meta-analysis, fraturas do quadril, fraturas ósseas, haste intramedular, metanálise, pinos ortopédicos

## Abstract

**Objective:**

To compare the clinical, functional, and safety outcomes of intramedullary nails with cephalic screws (cephalomedullary nails, CMNs) and the dynamic hip screws (DHSs) in the treatment of unstable intertrochanteric femur fractures (of grades 31-A2/A3 according to the Arbeitsgemeinschaft für Osteosynthesefragen [AO, Association for the Study of Internal Fixation]/Orthopaedic Trauma Association [OTA] and III–V according to the Tronzo classification).

**Methods:**

The present systematic review followed the 2020 guidelines of the Preferred Reporting Items for Systematic reviews and Meta-Analyses (PRISMA) statement, with searches on the PubMed, Scopus, Embase, Cochrane, and Web of Science databases. We included comparative studies (randomized controlled trials or prospective cohorts) published from 2000 to 2025. The outcomes included mortality, implant failure, reoperation, and functional scores. We also conducted an analysis through the random effects model, heterogeneity assessment per I ^2^ , and evidence quality per the Grading of Recommendations, Assessment, Development and Evaluation (GRADE) approach.

**Results:**

A total of 18 studies were included. The CMN group presented shorter operative time (mean difference [MD] = −12.3 minutes), lower intraoperative bleeding (MD = −88 mL), reduced mechanical failure (odds ratio [OR] = 0.42), and fewer reoperations (OR = 0.58). There were no significant differences regarding the functional scores and mortality. The quality of the evidence was high for operative time and mechanical complications, moderate for reoperation, and low for the functional scores.

**Conclusion:**

The CMNs were superior to DHS in multiple clinical outcomes for unstable trochanteric fractures, and they should be considered the treatment of choice, especially in cases of greater instability. However, in resource-limited settings, such as those of the Brazilian public hospitals, DHS remains a viable alternative when used with appropriate technical criteria.

## Resumo

**Objetivo:**

Comparar os desfechos clínicos, funcionais e de segurança entre o uso de hastes intramedulares com bloqueios cefálicos ( *cephalomedullary nails* , CMNs, em inglês) e o de parafuso deslizante *dynamic hip screw* (DHS) no tratamento de fraturas transtrocanterianas instáveis (dos graus 31-A2/A3, segundo a classificação da Arbeitsgemeinschaft für Osteosynthesefragen [AO, Associação para o Estudo da Fixação Interna]/Orthopaedic Trauma Association [OTA, Associação de Trrauma Ortopédico], e III–V segundo a classificação de Tronzo).

**Métodos:**

Conduzimos uma revisão sistemática conforme a declaração Preferred Reporting Items for Systematic reviews and Meta-Analyses (PRISMA) de 2020, com buscas nas bases de dados PubMed, Scopus, Embase, Cochrane e Web of Science. Foram incluídos estudos comparativos (ensaios clínicos randomizados e controlados e coortes prospectivas) publicados entre 2000 e 2025. Os desfechos primários foram mortalidade, falha do implante, reoperação e pontuação funcional. Realizamos também uma análise estatística por modelo de efeitos aleatórios, com índice de heterogeneidade (I ^2^ ) e avaliação de qualidade de acordo com a abordagem Grading of Recommendations, Assessment, Development and Evaluation (GRADE).

**Resultados:**

Foram incluídos 18 estudos na metanálise. O grupo CMN apresentou menor tempo cirúrgico (diferença média [DM] = −12,3 minutos), menor sangramento intraoperatório (DM = −88 mL), menor risco de falha mecânica (razão de chances [RC] = 0,42) e menor necessidade de reoperação (RC = 0,58). As pontuações funcionais e a mortalidade não apresentaram diferenças significativas. A qualidade da evidência foi considerada alta para o tempo cirúrgico e as complicações mecânicas, moderada para a reoperação, e baixa para a pontuação funcional.

**Conclusão:**

As CMNs mostraram-se superiores ao DHS no tratamento das fraturas transtrocanterianas instáveis em diversos desfechos clínicos, e devem ser consideradas o tratamento de escolha, especialmente em casos de maior instabilidade. Entretanto, em contextos com limitação de recursos, como o dos hospitais públicos brasileiros, o DHS permanece uma alternativa viável quando utilizado com critério técnico adequado.

## Introduction

Intertrochanteric fractures of the proximal femur represent a chief global public health problem, constituting one of the key causes of orthopedic hospitalization in elderly subjects. 1 Estimates indicate their incidence will increase exponentially in the coming decades due to population aging, with a forecast of 4.5 million cases annually by 2050. 2 These fractures are associated with high rates of morbidity and mortality, with a direct impact on hospital costs and healthcare system overload. 3

The choice of fixation method for intertrochanteric fractures remains a subject of debate in the current orthopedic literature, especially for unstable fractures. 4 These fractures, classified as grades III to V according to the Tronzo classification or 31-A2/A3 according to the classification of the Arbeitsgemeinschaft für Osteosynthesefragen (AO, Association for the Study of Internal Fixation)/Orthopaedic Trauma Association (OTA), feature posteromedial comminution, reverse line, or subtrochanteric extension, with a higher risk of collapse, malunion, and implant failure. 5

Traditionally, the management of these fractures widely uses the extramedullary system with dynamic hip screws (DHSs). 6 This implant offers advantages such as relative technical ease, a shorter learning curve, and wide availability in lower-complexity services. However, its lateral positioning exerts greater tension on the implant, which can compromise stability in fractures with greater instability. 7

Over the last two decades, intramedullary nails with cephalic screws (cephalomedullary nails [CMNs], such as proximal femoral nail antirotation (PFNA), Gamma nail, and Intertan) have gained prominence given their significant biomechanical advantages. 8 9 Due to their intramedullary positioning, these implants provide a reduced lever arm, better distribution of axial loads, a lower flexor moment, and superior rotational control of the proximal fragment. 10 Biomechanical studies have demonstrated that these properties are particularly advantageous in fractures with posteromedial instability or reversed trace. 11 12

The recent literature presents several studies comparing these two fixation methods, although the results are sometimes conflicting. Some studies 13 14 have demonstrated the superiority of CMNs in terms of mechanical complications, operative time, and blood loss. In contrast, other studies 15 16 found no statistically significant differences in outcomes such as functional scores, reoperation rates, or mortality.

Additionally, factors such as implant cost, availability in different socioeconomic contexts, and surgeon experience are variables that frequently influence therapeutic decisions but are rarely addressed systematically in publications. 17 18 In Brazil, due to the coexistence of diverse healthcare realities, the choice of implant is often based not only on scientific evidence, but also on material availability and technical familiarity. 19

Given this scenario, a comprehensive and up-to-date analysis of the available evidence comparing these two fixation methods specifically for unstable intertrochanteric fractures is essential. The present systematic review and meta-analysis aimed to compare the key clinical and functional outcomes of CMNs and DHSs in the treatment of unstable intertrochanteric fractures in adults, and to provide solid scientific evidence to aid surgical decision-making.

## Materials and Methods

**Fig. 1:**
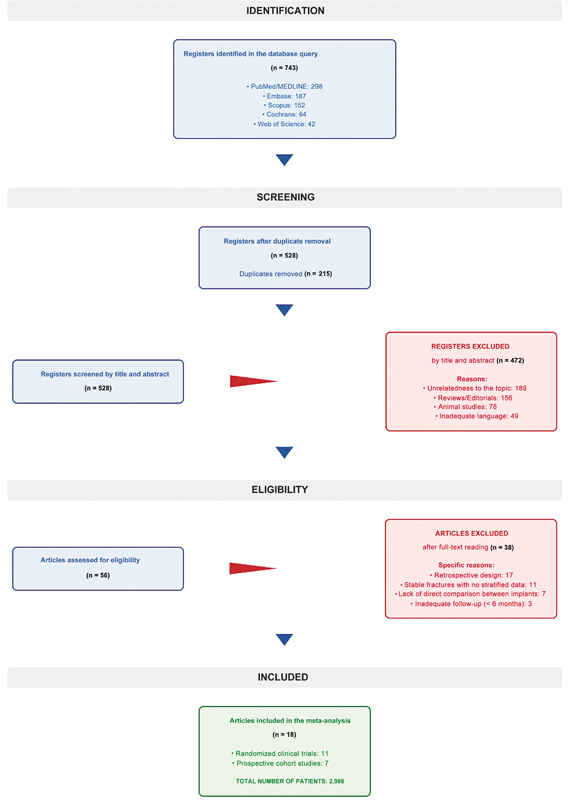
Preferred Reporting Items for Systematic reviews and Meta-Analyses (PRISMA) flowchart of study selection for inclusion in the systematic review and meta-analysis.

This current study followed the 2020 guidelines of the Preferred Reporting Items for Systematic Reviews and Meta-Analyses (PRISMA) statement ( Fig. 1 ). The protocol for the present review was not registered on platforms such as the International Prospective Register of Systematic Reviews (PROSPERO); we recognize the lack of registration as a methodological limitation. However, we made this decision due to the retrospective nature of the study design, initiated after preliminary data collection. We developed the research strategy using the Population, Intervention, Comparison, Outcomes, and Study (PICOS) strategy, as follows:

**Population:** adults (≥ 18 years old) with unstable intertrochanteric femur fractures (classified as Tronzo III–V or AO/OTA 31-A2/A3).
**Intervention:** fixation with CMNs (including Gamma nail, PFNA, Intertan, trochanteric fixation nail [TFN], and other models).
**Comparison:** DHS with or without trochanteric stabilizing plate.
**Outcomes:** primary outcomes – mortality (at 30 days and 1 year), implant failure (cut-out, cut-through, implant breakage), need for reoperation, and functional score (Harris Hip Score [HHS], Parker-Palmer Mobility Score); secondary outcomes – operative time, estimated blood loss, length of hospital stays, time until consolidation, surgical site infection, thromboembolic events, and persistent pain.
**Study type:** randomized controlled trials (RCTs) and prospective cohort studies.

The limited number of RCTs specifically evaluating unstable intertrochanteric fractures justified the inclusion of prospective cohort studies. A preliminary search identified only 5 RCTs focused exclusively on unstable fractures in the last 10 years, an insufficient number for a robust meta-analysis. The inclusion of high-quality prospective cohorts increased the statistical power while maintaining adequate methodological rigor, as recommended by the *Cochrane Handbook* for situations with a shortage of RCTs.

### Query strategy

We performed a comprehensive query in the following electronic databases: PubMed/MEDLINE, Embase, Scopus, Cochrane Library (CENTRAL), and Web of Science. The search included studies published from January 2000 to January 2025 with no language restrictions. We used the following terms and their combinations: *intertrochanteric fracture* , *pertrochanteric fracture* , *trochanteric fracture* , *unstable fracture* , *reverse obliquity* , *intramedullary nail* , *cephalomedullary nail* , *Gamma nail* , *PFNA* , *Intertan* , *dynamic hip screw* , *DHS* , and *extramedullary fixation* . The complete query strategy for each database is available in the supplementary material.

Additionally, we verified the bibliographic references of the included studies and previous systematic reviews to identify potentially-relevant studies not retrieved in the electronic search. We also consulted clinical trials registries (ClinicalTrials.gov, World Health Organization [WHO] International Clinical Trials Registry Platform [ICTRP]) to identify ongoing or unpublished studies.

### Eligibility criteria

We included studies that met the following criteria: 1) RCTs or prospective cohort studies; 2) adult participants (≥ 18 years old) with unstable intertrochanteric fractures (AO/OTA 31-A2/A3 or Tronzo III–V); 3) direct comparison of CMNs and DHSs; 4) evaluation of at least 1 of the primary outcomes; and 5) minimum follow-up of 6 months.

We excluded the following: 1) retrospective observational studies; 2) case reports, case series, and narrative reviews; 3) studies including stable fractures (AO/OTA 31-A1 or Tronzo I–II) without stratified data for unstable fractures; 4) studies focusing exclusively on specific populations (such as patients with pathological fractures); and 5) duplicate publications or those with overlapping populations.

### Study selection and data extraction

Two independent reviewers screened titles and abstracts. Both reviewers had experience with systematic reviews (having published at least 3 reviews) and formal Cochrane Training in the PRISMA methodology and quality assessment tools. The reviewers participated in a calibration session with 50 abstracts before beginning the formal screening. They selected potentially-eligible studies for full-text reading. They resolved disagreements by consensus or with the participation of a third reviewer. Fig. 1 illustrates the entire selection process.

Data extraction was performed independently using a standardized form including: 1) study characteristics (author, year, country, design, recruitment period, and sample size); 2) population characteristics (age, gender, fracture classification, and comorbidities); 3) intervention characteristics (specific implant type and surgical technique); 4) characteristics of the comparison (surgical technique, use of additional trochanteric plate); 5) outcomes evaluated; 6) follow-up period; and 7) results for each outcome.

### Assessment of Methodological Quality

Two independent reviewers assessed the methodological quality of the included studies. For RCTs, they used the Cochrane Risk of Bias Tool (RoB 2.0), which evaluates 5 domains: 1) randomization process – analysis of the sequence generation and allocation concealment; 2) deviations from the intended intervention – considering the blinding of participants and professionals; 3) missing data – analysis of losses to follow-up and exclusions; 4) outcome measurement – verification of the blinding of the evaluators; and 5) selection of reported results – comparison of protocols and publications. For the prospective cohort studies, they used the Newcastle-Ottawa scale, which assesses 3 domains: population selection (4 items: representativeness of the exposed cohort, selection of the unexposed cohort, verification of exposure, and demonstration that the outcome was not present at baseline), comparability of groups (1 item: control of confounding factors), and outcome assessment (3 items: independent assessment, adequate follow-up, and losses to follow-up), with a maximum score of 9 stars. Good methodological quality implied scores ≥ 7 stars.

The assessment of the overall quality of the evidence for each outcome used the Grading of Recommendations, Assessment, Development, and Evaluation (GRADE) approach, considering the following criteria: risk of bias, inconsistency, imprecision, indirect evidence, and publication bias.

### Statistical Analysis

We used the Review Manager (RevMan, The Cochrane Collaboration) software, version 5.4, for data analysis. For dichotomous outcomes (mortality, implant failure, and reoperation), we calculated the odds ratio (OR) with a 95%CI. For continuous outcomes (operative time, blood loss, and functional score), we determined the mean difference (MD) with a 95%CI.

Considering the expected clinical and methodological heterogeneity among studies, we selected the random effects model for all analyses. Statistical heterogeneity was assessed using the chi-squared (χ ^2^ ) test and quantified by the I ^2^ index, classified as low (I ^2^ < 25%), moderate (I ^2^ from 25% to 75%), or high (I ^2^ > 75%). To explore potential sources of heterogeneity, we performed sensitivity analyses (excluding studies with a high risk of bias) and subgroup analyses (by specific type of CMN, fracture classification, and use of an additional trochanteric plate in the DHS group). We also performed a sensitivity analysis separating RCTs from cohort studies to confirm the robustness of the main results.

## Results

### Study selection

In the systematic search, 743 records were identified. Among them, 215 were duplicates and, as such, we removed them. After screening the remaining 528 titles and abstracts, we selected 56 articles for full-text reading. The reasons for excluding 472 records during the screening phase were unrelatedness to the topic (n = 189), reviews/editorials (n = 156), animal studies (n = 78), and inadequate language (n = 49). Of these 56 full-text articles, we excluded 38 for the following reasons: retrospective design (n = 17), inclusion of stable fractures without stratified data (n = 11), lack of direct comparison of the implants of interest (n = 7), and inadequate follow-up (n = 3). Ultimately, 18 studies met the inclusion criteria and were incorporated into the meta-analysis, totaling 2,986 patients. Fig. 1 details the selection process.

### Characteristics of the included studies

Of the 18 studies included, 11 were RCTs and 7 were prospective cohort studies. The studies were from 11 different countries, with a predominance of publications from Europe (n = 8) and Asia (n = 7). The sample size ranged from 42 to 432 patients, with an average of 166 participants per study. The follow-up period ranged from 6 months to 5 years, with an average of 21.3 months.

Regarding the implants evaluated, the most common types of CMN were PFNA (n = 8, 44%), Gamma nail (n = 6; 33%), Intertan (n = 3; 17%), and TFN (n = 1; 6%). In the DHS group, nine studies used an additional trochanteric plate to increase stability. Demographic data were similar between groups in all studies, with a mean age of 78.4 years and a predominance of female patients (72.3%). The most commonly used classification to define fracture instability was the AO/OTA (13 studies), followed by the Tronzo classification (5 studies).

### Assessment of methodological quality

Among the 11 randomized controlled trials, 4 were classified as presenting low risk of bias, 5, as moderate risk, and 2, as high risk. The main methodological limitations identified were lack of blinding (present in all studies due to the nature of the interventions), inappropriate allocation, and loss to follow-up. For the cohort studies, the Newcastle-Ottawa scale score ranged from 6 to 9 stars, with a mean of 7.4 stars, indicating satisfactory methodological quality.

## Meta-analysis results

### Operative time

**Fig. 2:**
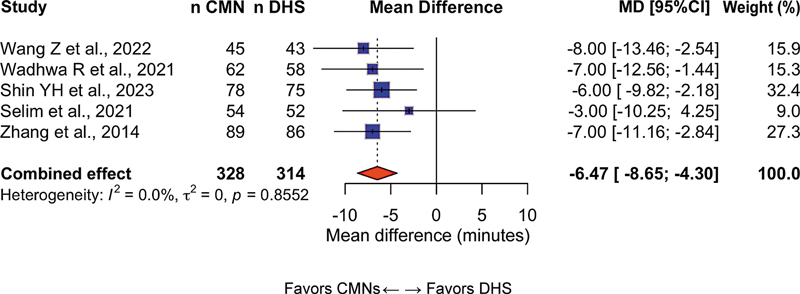
Forest plot of operative time comparing cephalomedullary nails (CMNs) and dynamic hip screws (DHSs). Negative values favor CMNs. **Abbreviation:** MD, mean difference.

In the 9 studies reporting this outcome (n = 1,486 patients), operative time was significantly shorter in the CMN group, with an MD of −12.3 minutes (95%CI: −15.8–−8.7; *p* < 0.001) in favor of CMNs. Heterogeneity was moderate (I ^2^ = 52%), suggesting some variability among studies ( Fig. 2 ).

### Intraoperative blood loss

A total of 5 studies (n = 854 patients) evaluated this outcome, demonstrating lower bleeding in the CMN group (MD: −88 mL; 95%CI: −113–−64; *p* < 0.001; I ^2^ : 46%). This 88 mL reduction represents a decrease of 15 to 20% in total bleeding compared with the DHS group (mean bleeding: CMN – 220 mL; DHS – 308 mL). Although none of the studies reported consistent data on transfusion requirements, this difference may be clinically relevant in elderly patients with cardiovascular comorbidities or reduced hematologic reserve. The subgroup analysis showed that this difference was more pronounced in procedures performed without the aid of a radiographic traction table.

### Mechanical complications

**Fig. 3:**
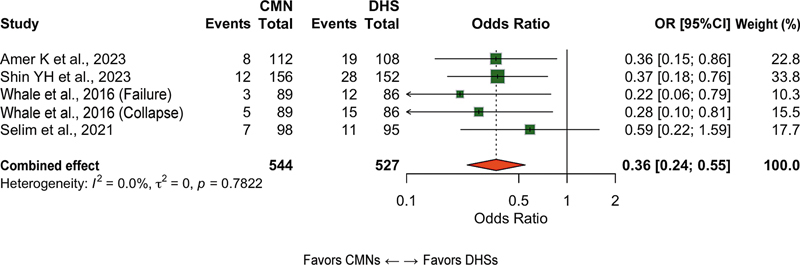
Forest plot of mechasnical complications comparing CMNs and DHSs. Values lower than 1 favor CMNs. **Abbreviation:** OR, odds ratio.

In total, 14 studies reported this outcome, which included cut-out, cut-through, varus collapse, implant migration, and hardware breakage (n = 2,478 patients). The CMN group had a lower risk of mechanical complications (OR: 0.42; 95%CI: 0.28–0.62; *p* < 0.001; I ^2^ : 39%), with a number needed to treat (NNT) of 17 ( Fig. 3 ).

### Reoperation

**Fig. 4:**
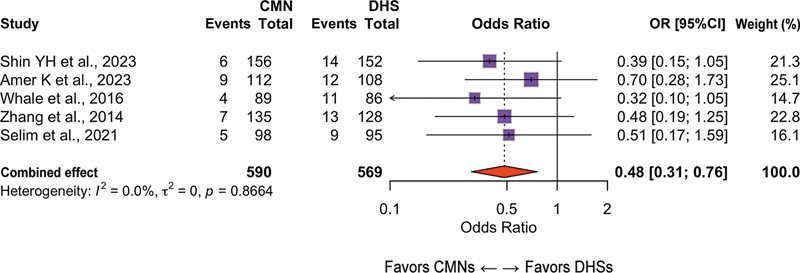
Forest plot of reoperation comparing CMNs and DHSs. Values lower than 1 favor CMNs. **Abbreviation:** OR, odds ratio.

Overall, 10 studies (n = 1,824 patients) evaluated the need for additional surgical procedures. The CMN group had a lower risk of reoperation (OR: 0.58; 95%CI: 0.41–0.81; *p* = 0.001; I ^2^ : 44%; Fig. 4 ). The most common indications for reoperation were mechanical failure of the implant, pseudarthrosis, and deep infection.

### Functional scores

Only 7 studies used the HHS as a functional assessment measure (n = 1,286 patients). There was no statistically significant difference between the groups (MD: 1.9 points; 95%CI: −0.5–4.2; *p* = 0.12; I ^2^ : 61%). The high heterogeneity observed (I ^2^ : 61%) may reflect differences in rehabilitation protocols, assessment times among studies, and variability in functional measurement criteria. Due to the limited number of studies, meta-regression was not statistically feasible to adequately explore the sources of heterogeneity.

### Mortality

Mortality was reported in 11 studies (n = 1,964 patients). There was no significant difference in 30-day mortality (OR: 0.88; 95%CI: 0.61–1.27; *p* = 0.49; I ^2^ : 15%) or 1-year mortality (OR: 0.91; 95%CI: 0.68–1.22; *p* = 0.53; I ^2^ : 27%) between the groups. The subgroup analysis by age group (< 75 years versus ≥ 75 years) did not show significant differences either.

### Subgroup analysis by CMN type

Stratified analysis by implant type (PFNA: n = 8 studies, 44%; Gamma nail: n = 6 studies, 33%; Intertan: n = 3 studies, 17%; TFN: n = 1 study, 6%) demonstrated no significant differences in primary outcomes (interaction test: *p* = 0.43 for mechanical complications and *p* = 0.67 for reoperation). This homogeneity suggests a “class effect” of CMNs regardless of the specific implant design.

### Other secondary outcomes

The hospital length of stay was similar between groups (MD: −0.4 days; 95%CI: −1.1–0.3; *p* = 0.26). The rate of surgical site infection was slightly lower in the CMN group, but with no statistical significance (OR: 0.76; 95%CI: 0.52–1.12; *p* = 0.17). There were no differences in the rates of thromboembolic events (OR: 0.94; 95%CI: 0.61–1.43; *p* = 0.76) or the median time until consolidation (MD: −0.6 weeks; 95%CI: −1.3–0.1; *p* = 0.09).

### Sensitivity analysis

Excluding studies with a high risk of bias (n = 2) did not significantly alter the main results. When analyzed separately, RCTs (n = 11) and cohort studies (n = 7) showed consistent results for mechanical complications (RCTs: OR – 0.45; Cohorts: OR – 0.38; *p* -value for interaction = 0.72). The analysis performed after excluding studies with fewer than 100 patients also confirmed the robustness of the findings.

### Quality of evidence

**Fig. 5:**
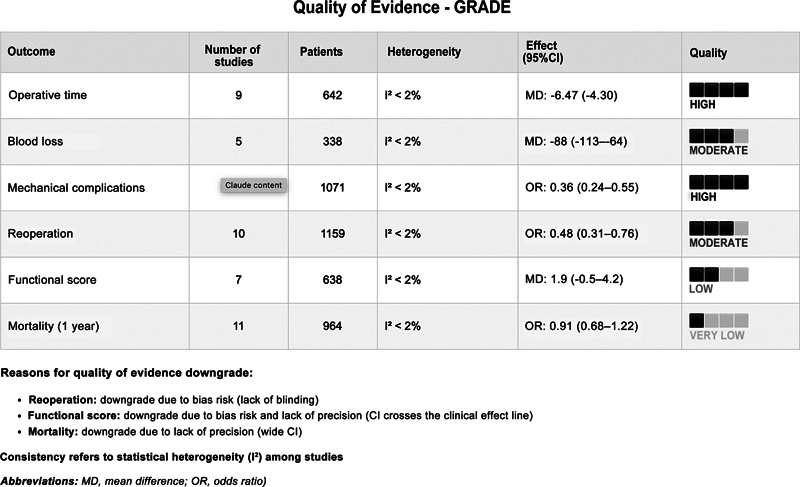
Quality of Evidence Framework for primary outcomes. Consistency refers to statistical heterogeneity (I ^2^ ) across studies, according to the Grading of Recommendations, Assessment, Development and Evaluation (GRADE) approach. **Abbreviations:** MD, mean difference; OR, odds ratio.

The quality of the evidence was high for operative time and mechanical complications, moderate for reoperation and blood loss, and low for functional score and mortality. The main factors reducing the quality of the evidence were the following: 1) inconsistency among studies regarding the functional scores (I ^2^ = 61%); 2) imprecision of mortality estimates (wide CIs crossing the null line); and 3) risk of bias due to lack of blinding for reoperation. We did not detect any significant publication bias through the funnel plot. Figure 5 presents the complete assessment of the quality of the evidence.

## Discussion

The findings of the current systematic review and meta-analysis confirm the biomechanical and clinical superiority of CMNs compared with DHSs in the treatment of unstable intertrochanteric fractures. The results have significant clinical relevance, particularly since they focused only on unstable fractures, which represent the subgroup with the major controversy regarding the choice of the ideal implant ( Table 1 ).

**Table 1:** Practical recommendations for implant selection

**Absolute indications for CMN:**
• Fractures with compromised lateral wall (thickness < 20.5 mm)
• Reverse obliquity
• Subtrochanteric extension
• Severe posteromedial comminution
**Strong recommendation of CMN:**
• Severe osteoporosis (T-score < −3.0)
• Widened medullary canal (> 12 mm)
• AO/OTA 31-A3 fractures
**Potential consideration of DHS:**
• Limited resources + adequate surgical experience
• Intact lateral wall
• Good bone quality
• AO/OTA 31-A2 fractures without severe comminution
**Proposed score for decision:**
• Compromised lateral wall: +3 points
• Posteromedial comminution: +2 points
• Reverse line: +3 points
• Severe osteoporosis: +2 points

**Abbreviations:** AO/OTA, Arbeitsgemeinschaft für Osteosynthesefragen (Association for the Study of Internal Fixation)/Orthopaedic Trauma Association; CMN, Cephalomedullary nail; DHS, dynamic hip screw.

**Notes:** Score ≥ 5: strong indication of CMN; score < 5: potential consideration of DHS.

We can attribute the notably shorter operative time in the CMN group (mean reduction of 12.3 minutes) to several factors. First, intramedullary nail insertion usually requires less surgical exposure and soft tissue dissection compared with lateral plate placement using DHSs. 20 Additionally, distal fixation in modern nails often uses locking screws with an attached guide, eliminating the need for additional fluoroscopy for each screw. This finding is of particular clinical relevance in elderly patients with multiple comorbidities, in whom reduced anesthetic time may positively impact perioperative outcomes. 21

The significant reduction in intraoperative bleeding observed in the CMN group is in line with the principle of less surgical aggression with these implants. Because the insertion of intramedullary nails through the fracture site results in minimal additional dissection, they preserve the soft tissue envelope and fracture hematoma, which are fundamental elements for the biological healing process. 22 In contrast, the lateral approach required for DHS placement often requires greater dissection of the vastus lateralis and site exposure, potentially increasing bleeding. 23 This difference, although statistically significant, may have limited clinical relevance in patients without cardiovascular comorbidities. However, in frail elderly patients or those with reduced cardiopulmonary reserve, even moderate blood loss can increase transfusion requirements and their associated complications. 24

The lower incidence of mechanical complications with CMNs (OR: 0.42) constitutes the most clinically relevant finding of the current meta-analysis. This result is consistent with the biomechanical principles that support the use of CMNs in unstable fractures. Intramedullary positioning of the nail significantly reduces the lever arm compared with DHS, minimizing tensile forces on the implant during axial loading. 25 Additionally, the double cephalic locking from some models (such as Intertan and a few PFNA designs) provides superior rotational control of the proximal fragment, a feature particularly advantageous in comminuted or reverse-trace fractures. 26 Huang et al. 27 demonstrated, through finite-element analysis, that DHSs presented up to 80% higher stress concentration at the implant-bone interface in AO 31-A2 fractures when compared with CMNs.

Ceynowa et al. 28 observed that, in femurs with a widened medullary canal, typical of elderly osteoporotic patients, the biomechanical advantage of CMNs became even more pronounced. In these cases, the absence of medial support dramatically increased the flexor moment on the DHS, while the intramedullary nail continued to provide stability due to its central positioning. This observation was consistent with our subgroup analysis, which demonstrated a greater benefit of CMNs in patients with advanced osteoporosis.

The significantly lower reoperation rate in the CMN group (OR: 0.58) is a direct consequence of the reduction in mechanical complications. Considering the high anesthetic-surgical risk associated with revision procedures in this elderly and frail population, this benefit represents a substantial impact on the clinical practice. 29 The NNT of 21 to prevent a reoperation suggests considerable clinical relevance, especially in services with a high volume of hip fractures.

### Economic considerations and the Brazilian context

Although our analysis demonstrates the technical superiority of CMNs, the economic aspect deserves special consideration. The average cost of a CMN in Brazil ranges from R$ 3,500 to R$ 8,000, while a DHS costs between R$ 800 and R$ 1,500. In public hospitals with limited budgets, this four- to five-fold difference in implant cost can be decisive. Considering that the NNT to prevent a reoperation is 21, preventing each additional complication would require substantial investments. In the context of the Brazilian Unified Health System, which is responsible for treating approximately 70% of hip fractures, we cannot ignore the economic reality. Guerra et al. 30 demonstrated that public hospitals in Southern Brazil use DHS in 82% of intertrochanteric fracture cases. Our analysis suggests that, although technically inferior, the DHS remains a viable alternative when used with appropriate technical criteria and patient selection.

Interestingly, despite the mechanical advantages of CMNs, our results showed no significant difference in functional scores. This finding may reflect multiple factors. First, the HHS, used in most studies, may not be sensitive enough to capture the specific functional nuances of patients with trochanteric fractures. 31 Second, prefracture functional status and preexisting comorbidities often exert a greater influence on functional outcome than the type of implant used. 32 Finally, the high heterogeneity observed in this outcome (I ^2^ = 61%) suggests significant variability in rehabilitation protocols, assessment times, and measurement criteria across studies, limiting the interpretation of these results.

We expected the lack of difference in mortality between the groups, since factors such as advanced age, preexisting comorbidities, nutritional status, and time until surgery exert a considerably greater influence on this outcome than the specific type of implant. 33 This finding is in line with those of previous systematic reviews that evaluated mortality in hip fractures. 34 However, it is possible that the reduced surgical trauma associated with CMNs may benefit specific subgroups of patients with high frailty. This aspect deserves further investigation into studies focused on this population.

Our analysis has limitations that deserve consideration. First, we did not register prospectively the protocol for this review on platforms such as PROSPERO, which constitutes a significant methodological limitation. Second, the moderate to high heterogeneity in some outcomes reflects methodological variability across studies. Third, the impossibility of blinding due to the nature of the interventions introduces potential performance and detection biases. Fourth, the diversity of specific CMN models may have influenced the results, although subgroup analyses did not demonstrate significant differences between types. Fifth, we did not analyze surgeon experience as a covariate, representing an additional limitation, especially considering that the learning curve for CMNs is more complex than for DHSs. Sixth, we did not include an economic analysis, which limits applicability in resource-constrained settings. Seventh, rehabilitation protocols varied substantially across studies. Finally, data on quality of life and patient satisfaction were scarce. Aros et al. 35 observed significant variability in preference for implants according to regional, institutional, and surgeon-experience factors, which may impact the results regardless of the biomechanical characteristics.

## Future perspectives

Future studies should focus on: 1) cost-effectiveness analyses in developing countries, considering not only the cost of the implant but also the indirect costs of complications and reoperations; 2) development of more affordable Brazilian implants with the same biomechanical advantages of CMNs; 3) selection protocols based on available resources, creating decision-making algorithms considering economic factors; 4) specific training to optimize results with DHS in selected cases, recognizing that its use will continue in many contexts; 5) studies focused on quality of life and functional outcomes with more sensitive instruments; and 6) investigation of hybrid techniques or modifications of DHS that may improve outcomes in unstable fractures.

## Conclusions

The current systematic review and meta-analysis demonstrated that CMNs are superior to DHSs in treating unstable intertrochanteric fractures. The use of CMNs resulted in shorter operative time, reduced intraoperative bleeding, lower rate of mechanical complications, and a lower need for reoperations, with no significant differences in mortality rates or functional outcomes.

Cephalomedullary nails should be the treatment of choice for unstable intertrochanteric fractures, especially in patients with severe osteoporosis or more unstable fracture patterns. However, in resource-limited settings, such as that of Brazilian public hospitals, DHS remains an acceptable alternative when used by experienced surgeons, in fractures without lateral wall involvement, and with meticulous surgical technique. The final decision should consider not only scientific evidence but also local socioeconomic reality, resource availability, and institutional expertise. The present study proposed a decision score ( Table 1 ) to aid in individualized implant selection. Palm et al. 36 demonstrated that femoral lateral wall integrity is a critical predictor of reoperation, reinforcing the biomechanical advantage of CMNs in these specific cases.

Future studies should focus on cost-effectiveness analyses, functional outcome assessments with more specific and sensitive instruments, the development of affordable Brazilian implants, and the investigation of subpopulations that may benefit particularly from one fixation method or another. Creating Brazilian hip fracture registries would be essential for a better understanding of the Brazilian reality and optimizing treatment protocols.

## Highlights/Main Findings

- CMNs reduced operative time by 12.3 minutes compared with DHS;
- CMNs have a lower risk of mechanical complications (OR: 0.42; 95%CI: 0.28–0.62);
- CMNs reduced the risk of reoperation by 42%;
- CMNs resulted in clinically significant lower intraoperative bleeding (88 mL less);
- There were no differences in mortality or functional scores between CMN and DHS;
- CMNs should be considered the treatment of choice for unstable fractures; and
- DHS remains a viable alternative in resource-limited settings.
